# Characteristics of Carotid Artery Structure and Mechanical Function and Their Relationships with Aortopathy in Patients with Bicuspid Aortic Valves

**DOI:** 10.3389/fphys.2017.00622

**Published:** 2017-08-28

**Authors:** Mihyun Kim, Chi Young Shim, Seong-Chan You, In-Jeong Cho, Geu-Ru Hong, Jong-Won Ha, Namsik Chung

**Affiliations:** Division of Cardiology, Severance Cardiovascular Hospital, Yonsei University College of Medicine Seoul, South Korea

**Keywords:** bicuspid aortic valve, aortopathy, ultrasound, velocity vector imaging, carotid artery

## Abstract

Patients with a bicuspid aortic valve (BAV) often have proximal aortic dilatation and systemic vascular dysfunction. We hypothesized that BAV patients would have different carotid artery structural and functional characteristics compared to tricuspid aortic valve (TAV) patients. In 28 patients with surgically confirmed BAV and 27 patients with TAV, intima media thickness (IMT), number of plaques, fractional area change (FAC), global circumferential strain (GCS), and standard deviation of CS (SD-CS) in both common carotid arteries were assessed using duplex ultrasound and velocity vector imaging (VVI). Patients with BAV were younger and had less co-morbidity, but showed a significantly larger ascending aorta (43.3 ± 7.5 vs. 37.0 ± 6.2 mm, *p* < 0.001) and a higher prevalence of aortopathy (61 vs. 30%, *p* = 0.021) than those with TAV. BAV patients showed a significantly lower IMT and fewer plaques. Although FAC and GCS were not significantly different between the two groups, they tended to be lower in the BAV group when each group was divided into three subgroups according to age. There was a significant age-dependent increase in IMT and decreases in FAC and GCS in the TAV group (*p* = 0.005, *p* = 0.001, *p* = 0.002, respectively), but this phenomenon was not evident in the BAV group (*p* = 0.074, *p* = 0.248, *p* = 0.394, respectively). BAV patients with aortopathy showed a higher SD-CS than those without aortopathy (*p* = 0.040), reflecting disordered mechanical function. In conclusion, BAV patients have different carotid artery structure and function compared with TAV patients, suggesting intrinsic vascular abnormalities that are less affected by established cardiovascular risk factors and more strongly related to the presence of aortopathy.

## Introduction

Bicuspid aortic valve (BAV) is the most common congenital heart valve disease (Verma and Siu, 2014). Patients with BAV are more likely to have proximal aortic dilation and systemic vascular dysfunction such as endothelial dysfunction or arterial stiffness than patients with a tricuspid aortic valve (TAV; Ferencik and Pape, 2003; Tadros et al., 2009). Intrinsic pathology of accelerated degeneration of the aortic media and combined hemodynamic factors are considered to be the main contributors to aortic dilation (Fedak et al., 2002; Wilton and Jahangiri, 2006). Reduced arterial elasticity in any site of the aorta or carotid artery has been proven in patients with BAV because the intrinsic pathologic alterations are not confined to the proximal part of aorta but extend into the entire aorta (Grotenhuis et al., 2007; Bilen et al., 2012).

Assessment of carotid artery structure and function has been highlighted in patients with aortic valve (AV) disease because carotid atherosclerosis and degenerative AV disease have common risk factors and pathogenesis. However, the structural and functional characteristics of the carotid artery in patients with BAV compared to those with TAV have not been comprehensively demonstrated. Also, the association between carotid mechanical dysfunction and the presence of aortopathy is uncertain (Petrini et al., 2014). Recently, lower carotid strain and a higher carotid stiffness index were shown in patients with BAV than in healthy controls, especially when the patients have dilation of the aorta (Li et al., 2016). In another study examining IMT of the descending aorta, IMT of the descending aorta was not directly influenced by the presence of BAV. Age was the main determinant of the aortic IMT (Petrini et al., 2016).

In this study, we hypothesized that: (1) patients with BAV would present less atherosclerotic structural change in the carotid arteries than those with TAV, (2) patients with BAV would have a different carotid mechanical function reflecting intrinsic wall abnormalities, and (3) the presence of aortopathy in patients with BAV would affect the carotid artery mechanical function. To support our hypotheses, we comprehensively evaluated carotid arterial structure and function using either carotid artery Duplex ultrasound or velocity vector imaging (VVI) in patients with BAV, compared the findings with those in patients with TAV, and analyzed the data according to the presence of aortopathy.

## Methods

### Study subjects

Twenty-eight patients with BAV disease and 27 patients with TAV were performed carotid artery Duplex ultrasound as part of preoperative evaluation before AV surgery at Severance Cardiovascular Hospital in Yonsei University (Seoul, South Korea) from January 2015 to January 2016. Patients with left ventricular systolic dysfunction (left ventricular ejection fraction < 40%), rheumatic AV disease, arrhythmia, systemic inflammatory disease, and those who were not suitable for analysis by carotid VVI were excluded from the study. The patients' medical records were comprehensively reviewed regarding the following demographic and clinical risk factors: age, sex, hypertension, diabetes mellitus, dyslipidemia, and coronary artery disease (diagnosed as greater than 50% angiographic narrowing). According to valve dysfunction, the subjects were analyzed separately either in patients with severe AS or in patients with severe AR. Also, we divided the subjects into subgroups according to age: group 1 (<59 years), group 2 (60–69 years), and group 3 (≥70 years). Patients with BAV and those with TAV were also each categorized into two subgroups according to the presence of aortopathy (ascending aorta diameter >40 mm). This study was approved by our institutional ethics committee and complied with the Declaration of Helsinki. Written informed consent was exempt by the board as this study was a research involving the existing noninvasive images. Patient records/information was anonymized and de-identified prior to analysis.

### Echocardiography

Standard two-dimensional and Doppler measurements were performed at least 1 month before the AV surgery following current recommendations for the assessment of valvular stenosis or regurgitation (Baumgartner et al., 2009; Lancellotti et al., 2010). Congenital BAV was diagnosed when only two cusps were unequivocally identified in systole and diastole in the short-axis view, with a clear “fish mouth” appearance during systole, as described in a previous study (Lee et al., 2015). BAV morphology was classified into the following four types according to position and pattern of raphe and cusps: Type 1, one raphe with fusion of the left coronary and right coronary cusps; type 2, one raphe with fusion of the right coronary and non-coronary cusps; type 3, one raphe with fusion of the left coronary and non-coronary cusps; type 0, no raphe with two developed cusps. The severity of aortic stenosis (AS) or aortic regurgitation (AR) was assessed using an integrated approach. All measurements of the aorta were performed according to recommendations on the QRS complex of the electrocardiogram (Lang et al., 2005). The dimension of the sinus of Valsalva was measured perpendicular to the right and left (or none) aortic sinuses. The sinotubular junction was measured where the aortic sinuses meet the tubular aorta. The ascending aorta (AA) was measured ~2 cm distal to the sinotubular junction. Continuous-wave Doppler was used to measure the aortic transvalvular maximal velocities, and peak and mean gradients were calculated using the simplified Bernoulli equation. AV area was calculated using the continuity equation (velocity-time integral method). Stroke volume was calculated using the Doppler method as follows: 0.785 × (left ventricular outflow track diameter) ^2^ × left ventricular outflow track velocity-time integral (Lang et al., 2005).

### Carotid artery duplex ultrasound

Both carotid arteries were assessed using a high-resolution, real-time, 8-MHz linear scanner (Acuson SC2000^TM^ Ultrasound System, Siemens Medical Solutions, USA Inc. Mountain View, CA). Optimal longitudinal and cross-sectional B-mode images of the common carotid arteries (CCAs) proximal to the bifurcation were obtained and stored digitally. A preliminary scan of internal, external, and common carotid arteries was performed to evaluate the presence of plaques and/or stenosis. Plaque was defined as either intima-media thickness (IMT) >1.3 mm or the presence of focal thickening at least 50% greater than the neighboring site on initial ultrasound examination. The IMT was calculated from manual measurements at the far wall of the carotid artery 1 cm proximal to the carotid bulb of both CCAs from leading edge (lumen-intima) to leading edge (media-adventitia) during end diastole (Sillesen et al., 2012).

### Carotid artery velocity vector imaging

**Figure 1:**
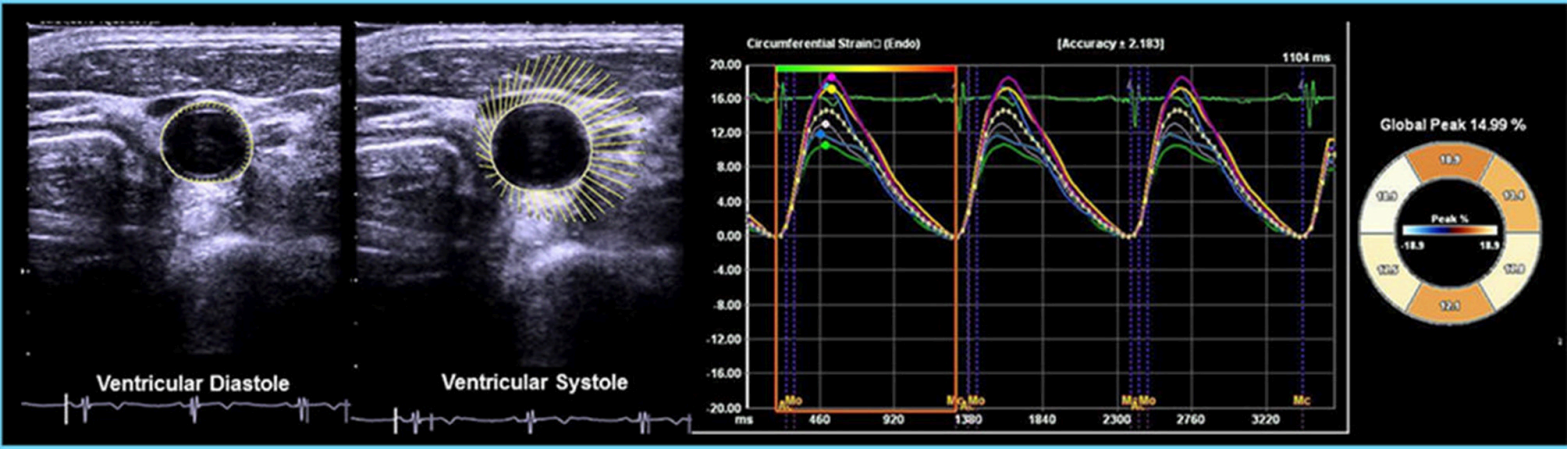
Representative example of VVI analysis of the carotid artery.

VVI measurements were performed on cross-sectional, two-dimensional ultrasound images of carotid arteries using an offline VVI workstation (Syngo US Workplace, Siemens). To obtain the optimal image for VVI analysis the images were stored at a rate of 60–120 frames/s using acoustic capture. Cross-sectional images of the CCA proximal to the bifurcation were scanned at the site without plaques. Cross-sectional images of the CCA either on the left or on the right from proximal to the bifurcation were divided into six segments. All segments were plotted by manually locating the endpoints of each segment on media–adventitia borders. The carotid artery borders were then identified and outlined according to the locations of the endpoints computed by the workstation (Cho et al., 2010; Yang et al., 2010, 2011). The fractional area change (FAC) was defined as the percentage change in cross-sectional area (CSA) between systole and diastole: FAC (%) = (largest CSA − smallest CSA)/(largest CSA) × 100. Global circumferential strain (GCS) was calculated as the average of peak circumferential strain of the six segments from each CCA (Kim et al., 2013). The GCS values from both CCAs were then averaged. The standard deviations of CS (SD-CS) of the six segments were analyzed on each CCA, and then the values from both CCAs were averaged. The inter-observer and intra-observer variability values for the VVI analyses were previously evaluated using 20 random carotid images in our laboratory (Cho et al., 2010; Yang et al., 2010). Representative images are shown in Figure 1.

### Statistical analyses

Data are expressed as mean ± standard deviation or as frequency and percentage. Continuous variables were compared using Student's *t*-test or analysis of variance (ANOVA), and categorical variables were compared with Fisher's exact test or Chi-squared test. To evaluate the relationships between aging and various carotid arterial measurements, participants were divided into subgroups according to age followed by evaluation of age-related changes in arterial measurements by analysis of variance. *P* < 0.05 were considered statistically significant. Statistical analysis was performed using standard software (SPSS 22.0, SPSS Inc., Chicago, IL).

## Results

### Baseline characteristics

Table 1 displays baseline characteristics of the two groups. Patients with BAV were significantly younger than those with TAV. The BAV subjects had fewer co-morbidities such as hypertension, dyslipidemia, and coronary artery disease, but gender proportion, prevalence of diabetes mellitus, history of smoking, average blood pressure, heart rate and systemic vascular resistance were comparable between the two groups. There were no significant differences in medication histories.

**Table 1:** Baseline characteristics of the study population.

	**BAV (*n* = 28)**	**TAV (*n* = 27)**	***p*-value**
Age, year	58.2 ± 11.1	70.2 ± 11.3	< 0.001
Men, *n* (%)	20 (71)	16 (59)	0.343
Body mass index, g/m^2^	24.0 ± 3.3	24.0 ± 2.6	0.962
Hypertension, *n* (%)	9 (32)	18 (67)	0.010
Diabetes mellitus, *n* (%)	1 (4)	4 (15)	0.147
Dyslipidemia, *n* (%)	12 (43)	18 (67)	0.038
Smoking, *n* (%)	12 (43)	7 (26)	0.187
CAD, *n* (%)	9 (33)	19 (70)	0.006
Systolic blood pressure, mmHg	120.4 ± 15.1	123.3 ± 12.8	0.445
Diastolic blood pressure, mmHg	73.6 ± 13.8	68.5 ± 8.5	0.103
Heart rate, bpm	67 ± 8	69 ± 12	0.475
Systemic vascular resistance, dyne-sec-cm^−5^	1416 ± 600	1354 ± 466	0.686
**MEDICATION**			
RAAS blocker, *n* (%)	13 (46)	18 (67)	0.250
ß-blocker, *n* (%)	8 (28)	12 (44)	0.316
Calcium channel blocker, *n* (%)	7 (25)	8 (30)	0.823
Diuretics, *n* (%)	11 (39)	19 (70)	0.055
Statin, *n* (%)	11 (39)	12 (44)	0.878

*BAV, bicuspid aortic valve; TAV, tricuspid aortic valve; CAD, coronary artery disease; bpm, beats per minute; RAAS, renin angiotensin aldosterone system*.

Table 2 shows echocardiographic characteristic of the two groups. In terms of AV dysfunction, about two-thirds of patients in each group had severe AS, and the remainder showed severe AR, without any differences between the two groups. According to the classification of BAV morphology, type 1 morphology was the most prevalent (*n* = 13, 45%). Patients with BAV had a significantly larger ascending aorta (43.3 ± 7.5 vs. 37.0 ± 6.2 mm, *p* < 0.001) and a higher prevalence of aortopathy (61 vs. 30%, *p* = 0.021) than those with TAV. The LV dimensions, ejection fraction, and stroke volume on echocardiography were comparable between the two groups. Regarding LV diastolic function, TAV patients had a higher E/e' and more enlarged left atrium than BAV patients.

**Table 2:** Echocardiographic characteristics of the study population.

	**BAV (*n* = 28)**	**TAV (*n* = 27)**	***p*-value**
**AORTIC VALVE DYSFUNCTION**			
Severe AS, *n* (%)	20 (71)	18 (67)	0.702
Severe AR, *n* (%)	8 (29)	9 (33)	
Mean systolic PG, mmHg	46.4 ± 23.6	50.1 ± 22.2	0.599
**BAV PHENOTYPES**			
Type 1 (RCC+LCC)	13 (45)	–	–
Type 2 (RCC+NCC)	5 (17)	–	–
Type 3 (LCC+NCC)	0 (0)	–	–
Type 0 (No raphe)	10 (34)	–	–
**AORTA CHARACTERISTICS**			
Valsalva sinus, mm	35.8 ± 8.1	33.9 ± 5.7	0.320
Ascending aorta, mm	43.3 ± 7.5	37.0 ± 6.2	< 0.001
Presence of aortopathy, n (%)	17 (61)	8 (30)	0.021
**LEFT VENTRICULAR FUNCTION**			
LVEDD, mm	53.3 ± 10.2	56.5 ± 11.7	0.275
LVESD, mm	36.1 ± 8.7	37.9 ± 10.4	0.495
LVEF, %	63.2 ± 7.8	64.4 ± 10.2	0.639
Stroke volume, ml	87.6 ± 28.9	86.2 ± 33.4	0.877
E/e'	13.1 ± 5.3	19.1 ± 9.8	0.026
LA volume index, ml/m^2^	38.9 ± 14.0	49.9 ± 17.5	0.008

*AS, aortic stenosis; AR, aortic regurgitation; PG, pressure gradient; LVEDD, left ventricular end-diastolic dimension; LVESD, left ventricular end-systolic dimension; LVEF, left ventricular ejection fraction; LA, left atrium*.

### Carotid artery structure and function

Table 3 shows the data from duplex ultrasound and VVI in the two groups. Patients with BAV showed a significantly lower IMT, fewer plaques, and a lower incidence of carotid artery stenosis. These structural differences probably reflect the younger age and lower prevalence of common cardiovascular risk factors and coronary artery disease in patients with BAV. In VVI with speckle tracking method, FAC and GCS, which reflect arterial distensibility and mechanical function, respectively, tended to be lower in patients with BAV compared to those with TAV even though the patients with BAV were younger.

**Table 3:** Carotid artery structure and function of the study population.

	**BAV (*n* = 28)**	**TAV (*n* = 27)**	***p*-value**
**DUPLEX ULTRASOUND**			
**Carotid intima-media thickness**			
Average, mm	0.77 ± 0.17	0.92 ± 0.16	0.003
Left, mm	0.79 ± 0.20	0.94 ± 0.20	0.005
Right, mm	0.76 ± 0.18	0.89 ± 0.19	0.012
Carotid plaque			
Total number	1.8 ± 2.0	3.2 ± 2.3	0.017
No plaque, *n* (%)	9 (32)	4 (15)	0.130
Single, *n* (%)	7 (25)	1 (4)	0.029
Multiple, *n* (%)	12 (43)	21 (81)	0.004
Carotid artery stenosis, *n* (%)	3 (11)	6 (22)	0.045
**VELOCITY VECTOR IMAGING**			
**Fractional area change**			
Average, %	8.52 ± 5.96	9.86 ± 6.25	0.421
Left, %	8.15 ± 6.12	9.36 ± 6.77	0.487
Right, %	8.90 ± 6.25	10.36 ± 6.26	0.392
**Peak circumferential strain, %**			
Average, %	3.67 ± 3.59	4.52 ± 3.52	0.380
Left, %	3.46 ± 3.71	4.22 ± 3.67	0.451
Right, %	3.88 ± 3.70	4.81 ± 3.77	0.354
***SD*** **of peak circumferential strain**			
Average, %	2.76 ± 1.90	3.21 ± 1.90	0.384
Left, %	2.27 ± 1.46	2.49 ± 1.27	0.553
Right, %	3.25 ± 3.06	3.93 ± 3.20	0.424

*BAV, bicuspid aortic valve; TAV, tricuspid aortic valve; SD, standard deviation*.

Table 4 presents the comparisons of carotid structure and function between BAV patients and TAV patients according to valve dysfunction. In patients with severe AS, number of carotid plaques tended to be lower in BAV patients than in TAV patients (*p* = 0.086). In the subgroup of severe AR, carotid IMT, number of carotid plaques and incidence of carotid artery stenosis were significantly lower in BAV patients compared to TAV patients (*p* = 0.019, *p* = 0.017, and *p* = 0.056, respectively). Although, the subgroups had a small number of patients, the main findings are consistent with the results from overall patients.

**Table 4:** Carotid artery structure and function in the subgroups according to valve dysfunction.

	**BAV**	**TAV**	***p*-value**
**With severe AS**	**(*****n*** = **20)**	**(*****n*** = **18)**	
Carotid intima-media thickness, mm	0.82 ± 0.17	0.89 ± 0.16	0.246
Carotid plaque, *n*	2.1 ± 2.1	3.5 ± 2.6	0.086
Carotid artery stenosis, *n* (%)	2 (10)	3 (17)	0.789
Fractional area change, %	6.17 ± 2.83	6.45 ± 2.44	0.731
Peak circumferential strain, %	2.39 ± 1.54	2.66 ± 1.13	0.567
SD of peak circumferential strain, %	3.57 ± 2.90	3.83 ± 2.14	0.696
**With severe AR**	**(*****n*** = **8)**	**(*****n*** = **9)**	
Carotid intima-media thickness, mm	0.68 ± 0.17	0.92 ±0.16	0.019
Carotid plaque, *n*	0.6 ± 0.8	2.5 ± 2.3	0.017
Carotid artery stenosis, *n* (%)	1 (13)	3 (33)	0.056
Fractional area change, %	13.79 ± 8.80	16.52 ±5.87	0.450
Peak circumferential strain, %	6.75 ± 5.29	4.52 ± 3.70	0.560
SD of peak circumferential strain, %	3.57 ± 2.90	3.83 ± 2.14	0.550

BAV, bicuspid aortic valve; TAV, tricuspid aortic valve; AS, aortic stenosis; AR, aortic regurgitation; SD, standard deviation

**Figure 2:**
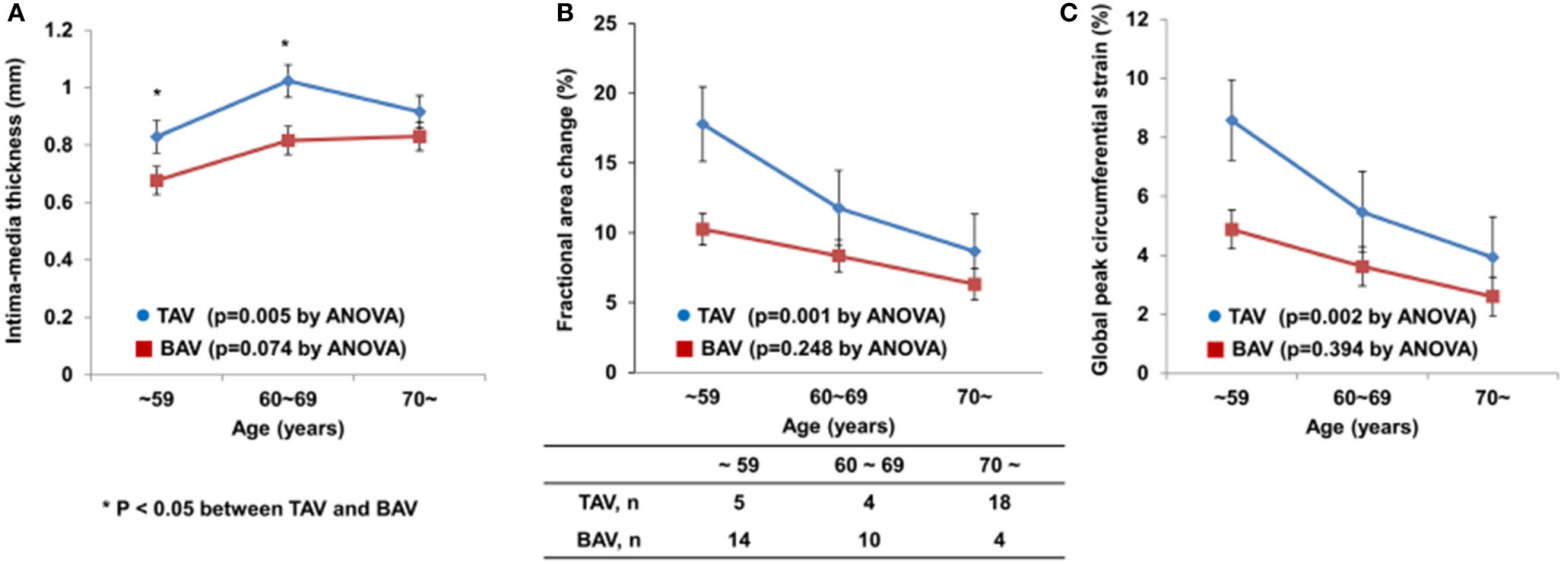
Age-dependent changes in carotid IMT **(A)**, FAC **(B)**, and GCS **(C)** in patients with BAV or TAV.

The patients in each group were classified into three subgroups according to age. Figure 2 shows carotid IMT, FAC, and GCS according to age group. Interestingly, there was a significant age-dependent increase in carotid IMT and decreases in FAC and GCS of CCAs in the TAV group (*p* = 0.005, *p* = 0.001, *p* = 0.002, respectively), but this phenomenon was not evident in the BAV group (*p* = 0.074, *p* = 0.248, *p* = 0.394, respectively). The average carotid IMT in patients with BAV who were < 70 years old was significantly lower than that in patients with TAV of corresponding age. In addition, FAC and GCS were consistently lower in patients with BAV than in patients with TAV in each subgroup based on age.

**Figure 3:**
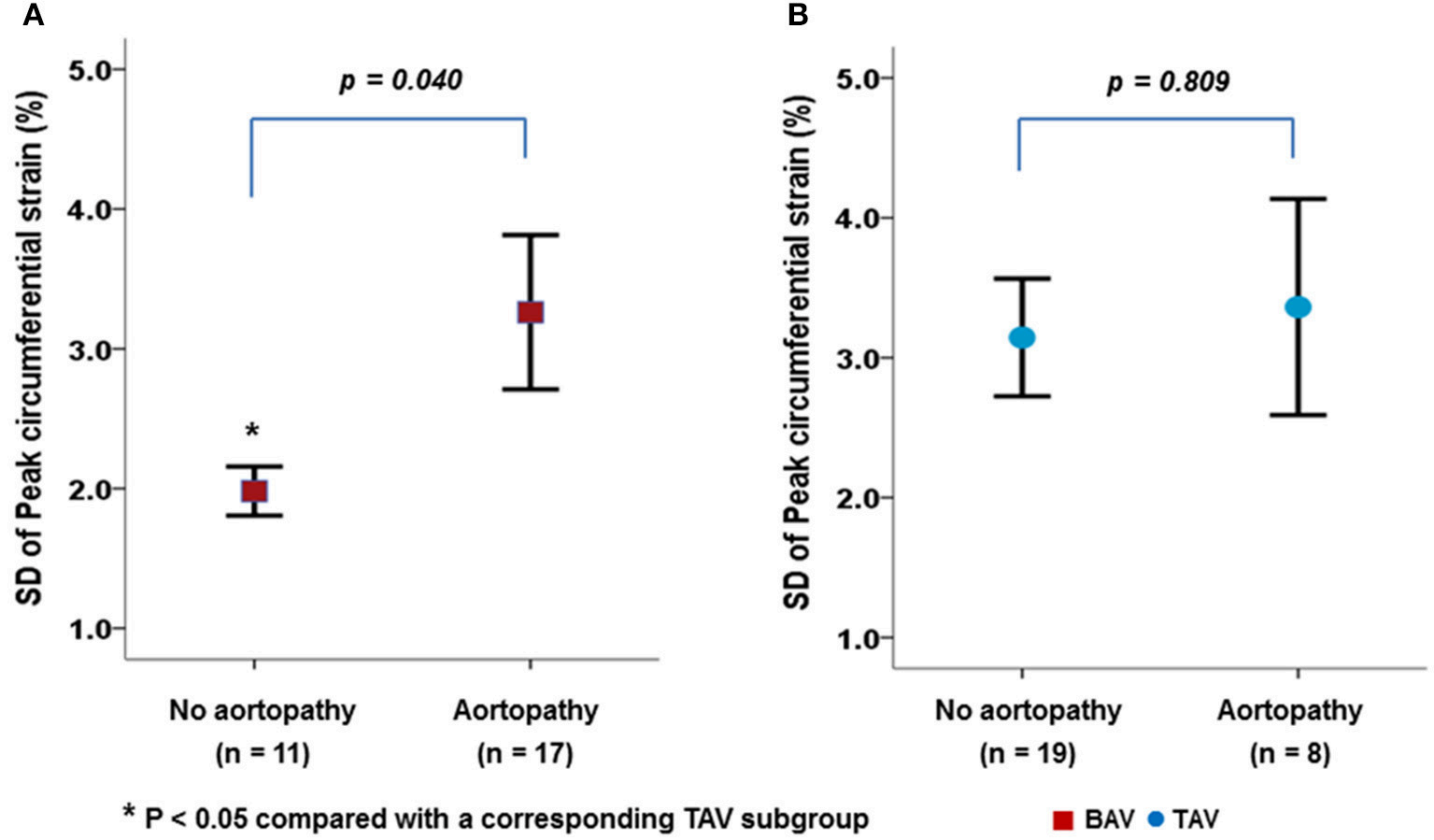
Differences in carotid mechanical function between BAV **(A)** and TAV **(B)** according to the presence of aortopathy.

Figure 3 presents the VVI parameters according to the presence of aortopathy in patients with BAV or TAV. BAV patients with aortopathy showed a significantly higher SD-CS than those without aortopathy (Figure 3A, *p* = 0.040). However, the presence of aortopathy had no apparent influence on carotid mechanical function in patients with TAV (Figure 3B, *p* = 0.809).

Table 5 presents the comparisons of aortic valve, aorta and carotid artery characteristics and function according to the BAV phenotypes. The number of carotid plaques was the highest in patients with type 0 phenotype probably due to the relatively higher prevalence of severe AS in the type 0 phenotype. The averages in FAC and GCS in patients with type 0 phenotype tended to be lower compared to those with other phenotypes without statistical significance.

**Table 5:** Aortic valve, aorta and carotid artery characteristics according to BAV phenotypes.

	**Type 1 RCC+LCC (*n* = 13)**	**Type 2 RCC+NCC (*n* = 5)**	**Type 0 No raphe (*n* = 10)**	***p*-value**
Age, year	54.9 ± 11.5	62.0 ± 9.4	61.0 ± 11.4	0.316
Severe AS, *n* (%)	8 (62)	3 (60)	9 (90)	0.051
Presence of aortopathy, *n* (%)	8 (62)	4 (80)	5 (50)	0.535
Carotid intima-media thickness, mm	0.73 ± 0.18	0.84 ± 0.20	0.83 ± 0.15	0.515
Carotid plaque, *n*	0.60 ± 0.91	2.20 ± 1.09	2.89 ± 2.66	0.009
Carotid artery stenosis, *n* (%)	1 (10)	2 (40)	1 (10)	0.689
Fractional area change, %	10.03 ± 7.88	7.67 ± 4.85	6.02 ±2.98	0.731
Peak circumferential strain, %	4.70 ± 4.69	3.04 ± 2.25	2.30 ± 1.62	0.277
SD of peak circumferential strain,%	3.51 ± 2.82	2.54 ± 0.65	2.31 ± 1.93	0.202

*BAV, bicuspid aortic valve; TAV, tricuspid aortic valve; AS, aortic stenosis; SD, standard deviation*.

## Discussion

The principal findings of this study are as follows: (1) Patients with BAV show fewer structural changes in carotid arteries related to atherosclerosis than patients with TAV, and (2) patients with BAV manifest greater mechanical dysfunction as assessed by VVI, probably related to arterial intrinsic pathology, compared to patients with TAV. Furthermore, our study reveals an important pathophysiological linkage between bicuspid aortopathy and vascular dysfunction by presenting the attenuated age-dependent changes in carotid arterial function and the relationship with the presence of aortopathy. To the best of our knowledge, this study is the first to demonstrate the characteristics of carotid artery structure and function in BAV patients with VVI.

### Histologic and functional changes of vasculature in patients with BAV

The BAV population has been reported to manifest increased degradation of elastin and collagen in the aortic wall mediated by matrix metalloproteinase-2, which is strongly expressed in tissue excised from the proximal aorta of patients with BAV (de Sa et al., 1999; Niwa et al., 2001). Overexpression of matrix metalloproteinase-2 is not only confined to the proximal aorta, but is also evident in the pulmonary trunk and thoracic aorta in patients with BAV (Fedak et al., 2005; Rabkin, 2014). In addition, a higher plasma level of matrix metalloproteinase-2 has been demonstrated in patients with BAV (Tzemos et al., 2010). The histological and serologic evidence of matrix metalloproteinase-2 overexpression in patients with BAV suggests a widespread pathology in systemic vasculature (Tzemos et al., 2010; Wang et al., 2014). On the other hand, BAV hemodynamics and flow abnormalities may initiate the aortopathy as a compensatory response to maintain constant wall shear stress. Abnormal hemodynamics in the ascending aorta may drive the production of circulating matrix metalloproteinase-2, and then affect the entire arterial system (Bissell et al., 2013).

Regarding systemic vascular function, increased central aortic stiffness in patients with BAV has been reported in previous studies (Nistri et al., 2008; Shim et al., 2011; Warner et al., 2013). Central aortic stiffness was positively correlated with the degree of aortic dilation in subjects with BAV (Shim et al., 2011). Interestingly, increased central aortic stiffness was found even in patients with non-dilated proximal aortas (Shim et al., 2011; Warner et al., 2013). Therefore, the functional changes of systemic arteries probably precede apparent structural changes. Recently, a few investigators have evaluated carotid elasticity using two dimensional and M-mode echocardiography (Li et al., 2016). The lower carotid strain and a higher carotid stiffness index in BAV patients with or without proximal aorta dilation than in healthy controls (Li et al., 2016). In another study examining IMT of the descending aorta, IMT of the descending aorta was not influenced by the presence of BAV but determined by aging (Petrini et al., 2016). Furthermore, the clinical implications of functional and structural abnormalities of the aorta have been highlighted in patients with BAV. In subjects with a normally functioning BAV, increased aortic stiffness and dilated ascending aorta were well correlated with left ventricular diastolic function (Lee et al., 2015). Moreover, in patients with moderately dysfunctional BAV, the presence of aortopathy and decreased aortic compliance were related to symptom status and clinical outcomes (Lee et al., 2017). Therefore, the assessment of structural and functional abnormalities of the proximal aorta and systemic vasculature is clinically important in patients with BAV.

In the present study, we evaluated vascular function using carotid VVI and presented different characteristics of carotid arterial mechanical function in patients with BAV compared to those with TAV. In general, the results from this study are consistent with the previous studies introduced above. Our findings suggest that the presence of aortopathy in a proximal ascending aorta in BAV patients is a marker of a more advanced process resulting in widespread alterations in artery elasticity (Fedak et al., 2002; Grotenhuis et al., 2007).

### Detection of vascular dysfunction using carotid VVI

Arterial assessment using VVI could represent a valuable new method for noninvasive quantification of vascular properties (Cannesson et al., 2006; Pirat et al., 2006). VVI allows direct assessment of angle-independent and instantaneous quantification of arterial elastic properties and vascular mechanics by measuring arterial wall strain and strain rate in both regional and segmental aspects (Kim et al., 2013). In previous studies using VVI, we characterized not only the vascular functional alterations in patients with specific vascular disorders such as Marfan syndrome or Takayasu's arteritis (Cho et al., 2010; Yang et al., 2010), but also normal vascular aging in healthy individuals (Yang et al., 2011). FAC, average CS, and strain rate in VVI were significantly correlated with conventional parameters of arterial stiffness. Moreover, the correlations between functional variables of VVI and collagen content of the arterial wall were validated in an animal experimental study (Kim et al., 2013). Since carotid duplex ultrasound is feasible and practical, it has been widely used for detection of subclinical atherosclerosis in clinical practice. The simple addition of VVI measurement for the carotid artery could provide incremental information for assessing vascular functional changes.

Many previous studies have demonstrated a marked reduction in arterial compliance with advancing age (Boutouyrie et al., 1992; Tanaka et al., 2000). We showed an age-dependent increase in carotid IMT and decreases in FAC and GCS of CCAs in patients with TAV, but this phenomenon was not evident in patients with BAV. These observations suggest that patients with BAV have intrinsic vascular abnormalities rather than features of common vascular aging that are affected by traditional cardiovascular risk factors. Recently, structural and functional assessment of carotid arteries with ultrasound has become popular not only for detection of subclinical atherosclerosis, but also for risk stratification before cardiac surgery. This study provides insight into the different carotid structure and function in patients with BAV. Therefore, to interpret the results from carotid VVI techniques, we should consider an individual's AV phenotype and the presence of aortopathy. Conversely, if there is difficulty in distinguishing the patient's AV phenotype because of extensive calcified AV, carotid artery structural and functional assessment might provide additional information.

## Limitations

Several limitations of this study should be noted. First, this study is a small size, cross-sectional study from a single center. Second, there was an age difference between the patients with BAV and those with TAV, although this was compensated by adjusting for age in the stepwise multivariate subgroup analysis. Second, all patients had significant AV dysfunction because they had been referred for carotid ultrasound as a part of preoperative carotid evaluation. Significant stenosis or regurgitation of the AV could influence carotid artery function. However, the proportion of patients with severe AS or AR and the mean systolic pressure gradient across the AV in each group were comparable. In addition, baseline left ventricular ejection fraction and stroke volume in each group were within normal ranges without any differences. Third, any circulating biomarkers relating BAV aortopathy were not assessed in the present study. Further studies are warranted for the correlation between circulating biomarkers and carotid mechanical function.

## Conclusion

Patients with BAV have different carotid artery structure and function compared to those with TAV, suggesting intrinsic vascular abnormalities that are less affected by traditional cardiovascular risk factors and aging and more strongly related to the presence of aortopathy. This finding supports the need for comprehensive evaluation of vascular function in patients with BAV.

## Author contributions

MK: Analyzed data; interpreted results; drafted manuscript; approved final version of manuscript; accountable for accuracy and integrity of the work. CS: Conception of research; design of research; analyzed data; interpreted results; prepared figures; reviewed and revised manuscript; approved final version of manuscript; accountable for accuracy and integrity of the work. SY: Design of research; analyzed data; IC: Edited and revised manuscript; GH: Conception of research; reviewed and revised manuscript; JH: Conception of research; edited and revised manuscript; NC: Edited and revised manuscript.

### Conflict of interest statement

The authors declare that the research was conducted in the absence of any commercial or financial relationships that could be construed as a potential conflict of interest.
